# ERAS Implementation Fidelity, Care Complexity, and Postoperative Outcomes in Oncological Colorectal Surgery: A Real-World Observational Study

**DOI:** 10.3390/healthcare14111519

**Published:** 2026-05-29

**Authors:** José Antonio Jerez González, Miguel Ángel Hidalgo-Blanco, Montserrat Puig Llobet, Jordi Adamuz, Maria Eulàlia Juvé-Udina, Oliver Polushkina-Merchanskaya, Bernat Miguel-Huguet, Mireia Mariscal Cabeza, Carmen Moreno Arroyo

**Affiliations:** 1 Bellvitge University Hospital, Faculty of Nursing, Bellvitge Biomedical Research Institute (IDIBELL), University of Barcelona and Nursing Research Group (GRIN), 08907 L’Hospitalet de Llobregat, Spain; jjerez@bellvitgehospital.cat; 2 Department of Fundamental and Clinical Nursing, Faculty of Nursing, Bellvitge Biomedical Research Institute (IDIBELL), University of Barcelona and Nursing Research Group (GRIN), 08907 L’Hospitalet de Llobregat, Spain; carmenmoreno@ub.edu; 3 Department of Public Health, Mental Health, and Maternal and Child Health Nursing, Faculty of Nursing, University of Barcelona, 08907 L’Hospitalet de Llobregat, Spain; monpuigllob@ub.edu; 4 Nursing Knowledge Management and Information Systems Department, Bellvitge University Hospital, Faculty of Nursing, Bellvitge Biomedical Research Institute (IDIBELL), Nursing Research Group (GRIN), University of Barcelona, 08907 L’Hospitalet de Llobregat, Spain; jadamuz@bellvitgehospital.cat; 5 Catalan Institute of Health, 08007 Barcelona, Spain; ejuve@gencat.cat; 6 Nursing Research Group (GRIN), Bellvitge Biomedical Research Institute (IDIBELL), 08907 L’Hospitalet de Llobregat, Spain; oliverpm327@gmail.com; 7 Data Management and Evaluation Unit, Bellvitge University Hospital, 08907 L’Hospitalet de Llobregat, Spain; bmhuguet.gtms.ics@gencat.cat; 8 Nursing Research Group (GRIN), Bellvitge Biomedical Research Institute (IDIBELL), Digestive Endoscopy Unit, Viladecans Hospital, 08840 Barcelona, Spain; mariscal.hv@gencat.cat

**Keywords:** ERAS, adherence, care complexity, perioperative care, colorectal surgery, healthcare delivery, length of stay

## Abstract

**Highlights:**

**What are the main findings?**

- Higher ERAS adherence is associated with shorter hospital length of stay.
- Adherence varies across perioperative phases, with lower compliance observed postoperatively.
- Care complexity does not show a significant association with outcomes.

**What are the implications of the main findings?**

- Findings highlight variability in ERAS implementation and support focusing on implementation fidelity in real-world clinical practice
- Results contribute to understanding ERAS as a healthcare delivery model, suggesting the need for further research on care complexity and recovery.

**Abstract:**

**Background:** Enhanced Recovery After Surgery (ERAS) programmes are structured perioperative care pathways in which clinical outcomes are closely linked to the degree of implementation fidelity. However, the interaction between ERAS adherence, care complexity, and postoperative outcomes in real-world settings remains insufficiently explored. **Objective:** To evaluate the association between ERAS adherence and postoperative length of stay in oncological colorectal surgery and to analyse whether Care Complexity Individual Factors (CCIFs) influence this relationship. **Methods:** A retrospective observational cohort study was conducted in two university hospitals in Barcelona, including 90 adult patients undergoing elective colorectal cancer surgery (2022). ERAS adherence was assessed globally and by phase (pre-, intra-, and postoperative) using structured indicators. CCIFs were classified into five domains. Associations between adherence, care complexity, and outcomes were analysed using bivariate methods. **Results:** Overall adherence was 64%. Higher adherence was associated with shorter hospital length of stay (median 4 vs. 5 days; *p* = 0.033) and greater compliance with expected length of stay (37.8% vs. 17.0%; *p* = 0.047). Adherence varied across perioperative phases, with higher compliance in the preoperative phase and lower compliance postoperatively. Care complexity was high (mean CCIF 2.62) and was not significantly associated with adherence or compliance with expected length of stay. **Conclusions:** Higher ERAS adherence is associated with shorter hospital stay in oncological colorectal surgery within a real-world context. These findings support the importance of implementation fidelity across the perioperative pathway. Further research incorporating multivariable analyses and patient-centred outcomes is needed to better understand the interaction between care complexity and recovery trajectories.

## 1. Introduction

Enhanced Recovery After Surgery (ERAS) programmes are structured, evidence-based perioperative care pathways designed to reduce surgical stress, optimise recovery, and improve clinical outcomes [1,2]. Their effectiveness has been consistently associated with the degree of adherence to protocol components across the preoperative, intraoperative, and postoperative phases [2,3,4,5,6,7].

Multiple studies, including large cohort analyses and meta-analyses, have demonstrated that higher compliance with ERAS protocols is associated with reduced postoperative complications, shorter hospital length of stay, and improved resource utilisation. This relationship has been described as a dose–response effect, in which incremental improvements in adherence translate into measurable clinical benefits [4,5,6,7].

Despite this evidence, ERAS implementation in real-world clinical practice remains variable [8,9], and adherence levels are often lower than those reported in controlled or highly standardised environments. This variability highlights the importance of examining ERAS not only as a clinical protocol but as a healthcare delivery model [9], in which organisational factors, professional engagement, and workflow integration play a central role [10].

At the same time, patients undergoing colorectal oncological surgery frequently present significant clinical and contextual complexity. Care Complexity Individual Factors (CCIFs) encompass a range of patient-related characteristics―including comorbidity burden, cognitive status, psychosocial conditions, and functional limitations―that may influence care processes and outcomes. While CCIFs have been associated with nursing workload and adverse events [11,12], their interaction with ERAS adherence has not been extensively studied.

This gap limits a comprehensive understanding of how implementation fidelity interacts with patient complexity in determining recovery trajectories. In particular, it remains unclear whether care complexity modifies the relationship between ERAS adherence and postoperative outcomes, or whether adherence itself remains the primary determinant of recovery in complex patient populations.

Therefore, the aim of this study is to evaluate the association between ERAS adherence and postoperative length of stay in oncological colorectal surgery, and to analyse whether Care Complexity Individual Factors influence this relationship in a real-world clinical context.

## 2. Materials and Methods

### 2.1. Study Design

A retrospective observational cohort study was conducted in two university hospitals in the province of Barcelona. Both centres follow similar colorectal surgical pathways and have implemented ERAS-based perioperative care protocols, although adherence levels vary in routine clinical practice.

Patients were followed from surgery until hospital discharge. ERAS adherence was analysed both globally and by phase (preoperative, intraoperative, and postoperative), based on structured indicators extracted from institutional databases.

### 2.2. Study Period and Population

Participants were identified from all colorectal surgical procedures performed between January and December 2022. Eligible patients were adults (≥18 years) undergoing elective colorectal surgery with a confirmed diagnosis of malignant neoplasm.

Exclusion criteria included emergency surgery, severe psychiatric conditions interfering with protocol adherence, and lack of sufficient social support to ensure safe discharge. In addition, as data were retrospectively collected from electronic health records, only patients with a documented electronic nursing care plan were eligible for inclusion, as this was required to assess Care Complexity Individual Factors (CCIFs). Patients without structured nursing documentation were therefore excluded.

A total of 132 patients were initially screened. After applying the inclusion and exclusion criteria, 90 patients were included in the final analysis.

Given the retrospective and exploratory nature of the study, no formal sample size calculation was performed a priori. However, a post hoc estimation indicated that, assuming an alpha risk of 0.05 and a power of 0.8 in a two-tailed test, a total of 88 subjects (44 per group) would be required to detect a difference of at least 3 days in length of stay, assuming a common standard deviation of 5. No drop-out rate was considered, as all available cases meeting inclusion criteria were included in the analysis.

### 2.3. Study Variables

#### 2.3.1. Sociodemographic and Clinical Variables

Sociodemographic and clinical data included age, sex, and comorbidities, as well as relevant clinical outcomes such as intensive care unit (ICU) admission and postoperative complications. Intensive care unit (ICU) admission refers in this context to a post-anaesthesia care or surgical recovery unit integrated within the critical care service. Admission to this unit may reflect not only clinical risk but also organisational factors, such as postoperative monitoring protocols or timing of surgery. In this study, ICU admission was considered a descriptive variable and was not included in analytical models.

#### 2.3.2. ERAS Adherence

ERAS adherence was assessed using structured indicators derived from institutional ERAS registries. Adherence was initially conceptualised as a continuous variable, expressed as a percentage (0–100%), and categorised into three levels: low (<50%), moderate (50–70%), and high (>70%). However, due to the structure and distribution of the available data, adherence was ultimately analysed as a dichotomous variable (adherent vs. non-adherent) for statistical comparison.

Global adherence was calculated as the mean of all protocol indicators, while phase-specific adherence scores were calculated separately for the preoperative, intraoperative, and postoperative phases. Importantly, postoperative adherence variables were interpreted with caution, as several of these indicators (such as oral intake or mobilisation) may reflect recovery capacity and therefore function as intermediate outcomes influenced by prior care delivery, rather than independent predictors (See Appendix A for the full list of items assessed in each phase).

To further explore the potential influence of postoperative adherence items on outcome measures, a sensitivity analysis excluding postoperative ERAS components was subsequently performed (Appendix F).

#### 2.3.3. Care Complexity Individual Factors (CCIFs)

Care complexity was assessed using the Care Complexity Individual Factors (CCIFs) framework described by Juvé-Udina et al. [11], which includes five domains: developmental, mental–cognitive, psycho-emotional, sociocultural, and comorbidity/complications. Each domain includes specific factors as previously described in the original CCIFs framework (Juvé-Udina et al. [11]). Each factor was recorded as present or absent for each patient, allowing the estimation of overall complexity burden as well as domain-specific contributions.

The detailed list of CCIFs domains and variables is provided in Appendix B.

#### 2.3.4. Postoperative Length of Stay

Postoperative Length of hospital Stay (PLOS) was defined as the number of days from surgery to hospital discharge, including ICU stay when applicable. The expected length of stay (EPLOS) was defined according to ERAS recommendations as 3 days for colon surgery and 4 days for rectal surgery.

Patients whose discharge was delayed due to non-clinical factors, such as social or organisational reasons, were not excluded but analysed within the overall cohort. No censoring was applied for transfers or in-hospital mortality, as these events were not present in the dataset.

Detailed ERAS indicators and CCIF variables are provided in Appendix A, Appendix B, Appendix C, Appendix D, Appendix E and Appendix F to enhance clarity and minimise potential overinterpretation given the limited sample size.

### 2.4. Data Collection Procedure

Data were obtained through structured extraction from multiple institutional sources, including electronic health records, nursing care plans using ATIC terminology, and ERAS indicator registries. Data extraction was performed by trained members of the research team and subsequently reviewed to ensure consistency and completeness.

Data cleaning procedures included the removal of duplicate records, validation of variable ranges, and resolution of discrepancies between data sources. Missing data were handled through case-wise exclusion for specific analyses in which incomplete variables were involved.

### 2.5. Statistical Analysis

Statistical analysis was performed using R software (version 4.4.1). Categorical variables were described using frequencies and percentages, while continuous variables were described using mean and standard deviation or median and interquartile range, depending on their distribution.

Comparisons between groups were conducted using Student’s *t*-test for normally distributed variables, the Kruskal–Wallis test for non-parametric data, and the chi-square test for categorical variables. Statistical significance was set at *p* < 0.05. Given the limited sample size, analyses were considered exploratory and hypothesis-generating, and no multivariable modelling was performed.

**Figure 1:**
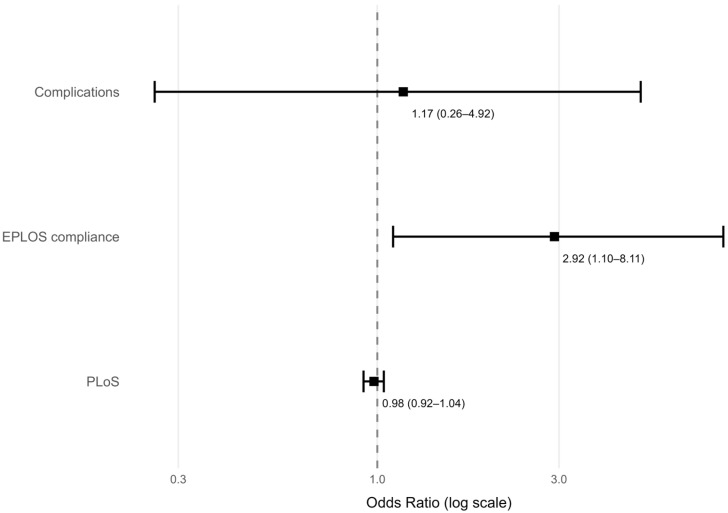
Forest plot showing the association between ERAS adherence and clinical outcomes. Odds ratios (OR) with 95% confidence intervals are presented. Values <1 indicate lower odds of the outcome in the adherent group. Estimates are unadjusted and should be interpreted cautiously.

A forest plot was generated to visually summarise the differences in key clinical outcomes between adherent and non-adherent patients, enhancing the interpretability of the results (Figure 1).

### 2.6. Ethical Considerations

The study was approved by the Clinical Research Ethics Committees of both participating hospitals (approval number: PR426/21). The study was conducted in accordance with the STROBE guidelines for observational studies [13], and the corresponding checklist is provided in Appendix C. Data were anonymised prior to analysis, and the requirement for informed consent was waived due to the retrospective nature of the study.

## 3. Results

### 3.1. Patient Characteristics and Care Complexity

The final study cohort included 90 patients who met the predefined inclusion criteria. The median age was 69.8 years [62.0; 76.2], and 57.8% were male. Most patients (88.9%) underwent surgery with curative intent, and the majority had a laparoscopic approach (71.1%).

Care complexity was high, with a mean of 2.62 CCIFs per patient. The most prevalent domains were comorbidity/complications (96.7%) and developmental factors (31.1%), followed by psycho-emotional and mental–cognitive domains (Table 1).

### 3.2. ERAS Adherence

Overall ERAS adherence was 64%, reflecting variable implementation across the cohort. Adherence differed by perioperative phase, with higher compliance observed in the preoperative phase compared to intraoperative and postoperative phases (Table 2).

### 3.3. Association Between ERAS Adherence and Outcomes

Higher ERAS adherence was associated with shorter hospital length of stay in the primary analysis. Patients classified as adherent had a median stay of 4.0 days compared to 5.0 days in the non-adherent group (*p* = 0.033). Table 3 summarises the association between ERAS adherence and key clinical outcomes.

Patients with higher overall ERAS adherence demonstrated a significantly greater likelihood of meeting the expected length of stay (37.8% vs. 17.0%, *p* = 0.047).

When analysing adherence across perioperative phases, higher compliance was observed in the preoperative phase compared to intraoperative and postoperative phases (Table 2). However, phase-specific associations with length of stay were not formally analysed in this study.

No statistically significant differences were observed in postoperative complications between groups (*p* = 0.105).

A sensitivity analysis excluding postoperative ERAS adherence items showed attenuation of the associations between adherence and postoperative outcomes, with no statistically significant differences observed for postoperative length of stay or EPLOS compliance (Appendix F).

A forest plot summarising the association between ERAS adherence and key clinical outcomes is presented in Figure 1. The plot suggests a trend towards shorter length of stay and improved EPLOS compliance in the adherent group, although confidence intervals indicate uncertainty for some outcomes.

### 3.4. Care Complexity and Outcomes

Table 4 summarises the association between care complexity and ERAS adherence as well as compliance with the expected length of stay (EPLOS).

No statistically significant association was observed between overall care complexity (CCIFs) and adherence levels or EPLOS compliance. Patients frequently presented multiple complexity factors; however, these did not appear to significantly influence adherence or recovery indicators in this cohort.

Similarly, no statistically significant differences were observed in the mean number of Care Complexity Individual Factors between patients who met and those who did not meet the expected length of stay (2.70 [SD 1.06] vs. 2.62 [SD 1.04]; *p* = 0.210).

A detailed breakdown of Care Complexity Individual Factors is provided in Appendix D and Appendix E.

## 4. Discussion

This study evaluated the relationship between ERAS adherence, care complexity, and postoperative outcomes in patients undergoing oncological colorectal surgery in a real-world clinical setting. The findings indicate that higher ERAS adherence is associated with shorter hospital length of stay [3,4,5,14], supporting the relevance of implementation fidelity in perioperative care pathways. The forest plot representation suggests a consistent direction of association across key outcomes, although estimates should be interpreted cautiously due to the width of confidence intervals.

Although phase-specific associations were not formally analysed, differences in adherence across perioperative phases may have implications for recovery, as suggested in previous ERAS literature [15,16]. This interpretation is further supported by the sensitivity analysis excluding postoperative adherence items, which attenuated the observed associations with postoperative outcomes.

Despite the high burden of care complexity observed in the cohort, no significant association was found between Care Complexity Individual Factors and postoperative outcomes. These findings suggest that, within this context, adherence to structured perioperative care pathways may be more closely associated with recovery than patient complexity alone [6,12]. However, this interpretation should be considered exploratory, given the limited sample size.

The variability in ERAS adherence observed in this study reflects the challenges of implementing complex care protocols in routine clinical practice [8,9]. While both participating centres had adopted ERAS-based pathways, overall adherence remained below optimal levels, highlighting the gap between protocol adoption and effective implementation. These findings support the view of ERAS not only as a clinical protocol but also as a healthcare delivery model [7,9] requiring organisational alignment and sustained engagement.

From a clinical perspective, the results suggest that efforts to improve perioperative outcomes should prioritise strengthening adherence across all phases of care. Although the potential contribution of nursing-led interventions to improving adherence is recognised in the literature [17], the present study does not allow causal inference in this regard and should be interpreted accordingly.

This study has several limitations. Its retrospective design and relatively small sample size limit the ability to perform multivariable analyses and may reduce statistical power for subgroup comparisons. The absence of multivariable analysis limits the ability to account for potential confounding factors such as age, comorbidities, and postoperative complications. Therefore, the observed associations should be interpreted as exploratory and hypothesis-generating. Furthermore, the use of convenience sampling and the requirement for structured electronic nursing documentation may have introduced selection bias.

Finally, the absence of 30-day follow-up, readmissions, and mortality data restricts the assessment of broader postoperative outcomes. Although care complexity was measured using a structured framework, the level of detail required to fully explore its impact may exceed the analytical capacity of the available sample.

## 5. Conclusions

Higher adherence to ERAS protocols was associated with shorter hospital length of stay in oncological colorectal surgery within a real-world clinical setting. Adherence varied across perioperative phases, although phase-specific associations with outcomes were not formally analysed in this study.

Care complexity did not show a statistically significant association with postoperative outcomes in this cohort; however, this finding should be interpreted cautiously given the exploratory nature of the study, the limited sample size, and the absence of multivariable adjustment.

These findings suggest an association between implementation fidelity and postoperative recovery, although the relative contribution of care complexity cannot be definitively established. Further research in larger cohorts using adjusted analytical approaches is needed to better understand these relationships.

## Figures and Tables

**Table 1:** Patient Characteristics and Care Complexity Profile.

	All
	*N* = 90
**Gender**	
Female	38 (42.2%)
Male	52 (57.8%)
**Age (median, [Q1; Q3])**	69.8 [62.0; 76.2]
**Surgical approach (laparoscopic)**	64 (71.1%)
**Surgical site, *n* (%)**	
Colon	62 (68.9%)
Rectal	28 (31.1%)
**ICU admission**	21 (23.3%)
**Complications**	9 (10.0%)
**Postoperative Length of Stay (PLoS)**	5.00 [4.00; 8.00]
**Expected PLoS**	23 (25.6%)
**CCIFs, mean (SD)**	2.62 (1.04)
**Domain**	
Mental–cognitive	3 (3.33%)
Psycho-emotional	11 (12.2%)
Sociocultural	1 (1.11%)
Developmental	28 (31.1%)
Comorbidities/Complications	87 (96.7%)

Values are presented as median (interquartile range, IQR), mean (standard deviation, SD), or number (percentage), as appropriate. CCIFs: Care Complexity Individual Factors. ICU: Intensive Care Unit (postoperative recovery unit in the context of this study).

**Table 2:** Global and Phase-Specific ERAS Adherence.

	All
Overall ERAS adherence, mean (SD)	0.64 (0.22)
Preoperative adherence, mean (SD)	0.86 (0.17)
Intraoperative adherence, mean (SD)	0.63 (0.27)
Postoperative adherence, mean (SD)	0.53 (0.31)

Values are expressed as mean (standard deviation, SD). ERAS adherence is reported as a proportion ranging from 0 to 1, corresponding to 0% to 100% compliance with protocol items. Phase-specific adherence refers to compliance within preoperative, intraoperative, and postoperative ERAS components.

**Table 3:** Association Between ERAS Adherence and Clinical Outcomes.

	All	Non-Adherent	Adherent	OR (95% CI)	*p*-Value
	*N* = 90	*N* = 53	*N* = 37		
**Postoperative Length of Stay (PLOS), median [Q1; Q3]**	5.00 [4.00; 8.00]	5.00 [5.00; 8.50]	4.00 [3.00; 4.00]	0.98 [0.92; 1.04]	0.033
**EPLOS compliance (%)**	23 (25.6%)	9 (17.0%)	14 (37.8%)	2.92 [1.10; 8.11]	0.047
**Complications, *n* (%)**	9 (10.0%)	5 (9.43%)	4 (10.8%)	1.17 [0.26; 4.92]	0.105

Values are presented as median (IQR) or number (%), as appropriate. Odds ratios (OR) with 95% confidence intervals are unadjusted and derived from bivariate analyses; they should be interpreted cautiously. EPLOS: Expected postoperative length of stay.

**Table 4:** Association Between Care Complexity (CCIFs), ERAS Adherence, and Compliance with Expected Length of Stay (EPLOS).

	**Non-Adherent**	**Adherent**	**OR** **(95% CI)**	***p*-Value**
	*N* = 53	*N* = 37		
**CCIFs, mean (SD)**	2.45 (0.99)	2.86 (1.08)	1.48 [0.97; 2.26]	0.070
**Domain**				
Mental–cognitive	2 (3.77%)	1 (2.70%)	0.75 [0.02; 9.65]	1.000
Psycho-emotional	1 (1.89%)	10 (27.0%)	16.7 [2.91; 429]	<0.001
Sociocultural	1 (1.89%)	0 (0.00%)		1.000
Developmental	18 (34.0%)	10 (27.0%)	0.73 [0.28; 1.82]	0.640
Comorbidities/Complications	51 (96.2%)	36 (97.3%)	1.33 [0.10; 42.7]	1.000
	**Non EPLOS Compliance**	**EPLOS Compliance**	**OR** **(95% CI)**	***p*** **-Value**
	*N* = 67	*N* = 23		
**CCIFs, mean (SD)**	2.70 (1.06)	2.39 (0.99)	0.74 [0.46; 1.19]	0.210
**Domain**				
Mental–cognitive	2 (2.99%)	1 (4.35%)	1.56 [0.05; 20.1]	1.000
Psycho-emotional	7 (10.4%)	4 (17.4%)	1.81 [0.42; 6.89]	0.462
Sociocultural	0 (0.00%)	1 (4.35%)	--	0.256
Developmental	22 (32.8%)	6 (26.1%)	0.73 [0.23; 2.07]	0.732
Comorbidities/Complications	65 (97.0%)	22 (95.7%)	0.64 [0.05; 20.8]	1.000

Values are presented as mean (standard deviation, SD) or number (%), as appropriate. Odds ratios (OR) with 95% confidence intervals are unadjusted and derived from bivariate analyses; they should be interpreted cautiously. CCIFs: Care Complexity Individual Factors; EPLOS: Expected postoperative length of stay.

## Data Availability

The data presented in this study are not publicly available due to ethical and privacy restrictions, as they contain sensitive information regarding individual participants and affiliated hospitals. Access to the data may be considered upon reasonable request and with appropriate ethical approval.

## Abbreviations

The following abbreviations are used in this manuscript:

CCIFs	Care Complexity Individual Factors
EPLOS	Expected postoperative length of stay
ERAS	Enhanced Recovery After Surgery
GRIN	Nursing Research Group
ICU	Intensive Care Unit
IDIBELL	Bellvitge Biomedical Research Institute
NURSEARCH	Nursing Research Group in Mental Health, Psychosocial and Complex Care
PLOS	Postoperative length of stay
SD	Standard deviation

## Appendix A. ERAS Programme Indicators and Operational Definitions

**Level**	**INDICATOR**	**Indicator Description**	**Variable Code**	**Variable Description Label**
1. Preoperative	IP01	Preoperative information	COMP_INFO	Understanding of the information provided
1. Preoperative	IP01	Preoperative information	GUIA_IN	Was the information booklet provided to the patient?
1. Preoperative	IP01	Preoperative information	APACI	Has the patient watched the video?
1. Preoperative	IP02	Preoperative assessment	FUMADOR	Smoker
1. Preoperative	IP02	Preoperative assessment	ACFIDOLA_L	Domestic/occupational physical activity
1. Preoperative	IP02	Preoperative assessment	ALCOHOLSN	Alcohol consumption (yes/no)
1. Preoperative	IP02	Preoperative assessment	CORRE	Anaemia correction
1. Preoperative	IP02	Preoperative assessment	ESC_MUST	MUST score (Malnutrition Universal Screening Tool)
1. Preoperative	IP02	Preoperative assessment	VALIMC	BMI assessment
1. Preoperative	IP03	Mechanical bowel preparation	PREP	Preparation
1. Preoperative	IP04	Preoperative fasting and carbohydrate loading	ING_LIQUI	Fluid intake up to 2 h before surgery
1. Preoperative	IP04	Preoperative fasting and carbohydrate loading	ING_SOLI	Solid food intake up to 6 h before surgery
1. Preoperative	IP04	Preoperative fasting and carbohydrate loading	PRE_CAR	Carbohydrate loading
1. Preoperative	IP04	Preoperative fasting and carbohydrate loading	NU_POT	Units of Maltodextrine consumed
2. Intraoperative	IP05	Thromboembolism prophylaxis	PRO_HBPM	Education on LMWH prophylaxis
2. Intraoperative	IP05	Thromboembolism prophylaxis	MIT_COM	Compression stockings/intermittent pneumatic compression
2. Intraoperative	IP06	Antibiotic prophylaxis	PRO_PREQ	Preoperative antibiotic prophylaxis
2. Intraoperative	IP07	Surgical approach	LAPARO	Laparoscopy
2. Intraoperative	IP08	Fluid management	BAL_LIQ_IQ	Intraoperative fluid balance
2. Intraoperative	IP08	Fluid management	RE_SUERO	Discontinuation of intravenous fluid therapy
2. Intraoperative	IP09	Prevention of hypothermia	MESURES	Physical measures for hypothermia prevention
3. Postoperative	IP10	Drains	NU_DRE	Number of abdominal wall drains
3. Postoperative	IP10	Drains	DRE_AB	Number of intra-abdominal drains
3. Postoperative	IP11	Oral medication	ANA_ORAL	Oral analgesia
3. Postoperative	IP12	Pain control	EVA	VAS score (Visual Analogue Scale)
3. Postoperative	IP13	Nutritional intake	SU_HIPERPR	Oral high-protein supplements
3. Postoperative	IP13	Nutritional intake	PROGRE_DIE	Diet progression
3. Postoperative	IP14	Early mobilisation	SED_6	Mobilisation at 6 h postoperatively
3. Postoperative	IP14	Early mobilisation	SED	Not bedbound

## Appendix B. Care Complexity Individual Factors (CCIFs) Domains and Variables

The Care Complexity Individual Factors (CCIFs) framework includes multiple domains reflecting patient-related complexity. Each domain comprises specific variables as detailed below.

**Domain**	**Factors**
**Mental–cognitive**	Agitation; Mental status impairments; Impaired cognitive function; Perception of reality disorders
**Psycho-emotional**	Aggressiveness; Fear/Anxiety; Impaired adaptation
**Sociocultural**	Language barriers; Social exclusion; Belief conflict; Illiteracy; Lack of caregiver
**Developmental**	Older age
**Comorbidities/Complications**	Major chronic disease; Haemodynamic instability; Risk of haemorrhage; Communication disorders; Urinary or faecal incontinence; Vascular fragility; Postural limitation; Involuntary movements; Extreme weight; Dehydration; Oedema; Uncontrolled pain; Transmissible infection; Immunosuppression; Anatomical-functional disorders

## Appendix C. STROBE Checklist for Observational Studies

This checklist follows the STROBE (Strengthening the Reporting of Observational Studies in Epidemiology) guidelines and indicates where each recommendation is addressed in the manuscript.

**Section**	**Item**	**Recommendation**	**Reported in Manuscript**
**Title and Abstract**	1	Indicate the study design in the title or abstract	Title and Abstract
		Provide an informative and balanced summary	Abstract
**Introduction**	2	Explain scientific background and rationale	Introduction
	3	State specific objectives	End of Introduction
**Methods**	4	Present key elements of study design	Section 2.1
	5	Describe setting, locations, and relevant dates	Section 2.2
	6	Give eligibility criteria and sources of participants	Section 2.2
		Describe selection methods	Section 2.2
	7	Clearly define all outcomes and variables	Section 2.3
	8	Describe data sources and measurement methods	Section 2.3 and Section 2.4
	9	Describe efforts to address bias	Section 2.3.2 and Section 2.5
	10	Explain how study size was determined	Section 2.2 (retrospective inclusion)
	11	Explain handling of quantitative variables	Section 2.3.2 and Section 2.5
	12	Describe statistical methods	Section 2.5
		Methods to examine subgroups and interactions	Section 2.5
		Explain how missing data were addressed	Section 2.4
**Results**	13	Report number of individuals at each stage	Section 2.2/Results
		Reasons for non-participation	Section 2.2
		Consider use of flow diagram	(Optional―not included)
	14	Give characteristics of study participants	Results
		Indicate missing data for each variable	Results
	15	Report outcome data	Results
	16	Provide main results with estimates and precision	Results
		Adjusted estimates (if applicable)	Not applicable (no multivariable analysis)
	17	Report other analyses (subgroups, sensitivity)	Results
**Discussion**	18	Summarise key results	Discussion
	19	Discuss limitations	Discussion
	20	Provide cautious interpretation	Discussion
	21	Discuss generalisability	Discussion
**Other Information**	22	Give source of funding and role of funders	Funding

## Appendix D. Numbers of CCIFs in All Perioperative Phases

**Perioperative Phase**	**Patients Adhered**	**Patients Not Adhered**	***p*-Value**
Overall Adherence	2.86 (1.08)	2.45 (0.99)	0.070
Preoperative phase	2.66 (1.03)	2.30 (1.16)	0.366
Intraoperative phase	2.59 (1.02)	2.67 (1.10)	0.748
Postoperative phase	2.83 (1.15)	2.52 (0.98)	0.202

## Appendix E. Association Between CCIFs and Compliance with Expected Length of Stay (EPLOS)

**CCIFs**	**EPLOS**				
	**Yes (N = 23)**		**No (N = 67)**		***p*-Value**
	**N**	**(%)**	**N**	**(%)**	
**Number of factors―Mean (SD)**	2.70	(1.06)	2.62	(1.04)	0.210
**Mental–cognitive**	1	(4.35)	2	(2.99)	1.000
Agitation	0	(0.00)	0	(0.00)	
Mental status impairments	1	(4.35)	2	(2.99)	1.000
Impaired cognitive function	0	(0.00)	0	(0.00)	
Perception of reality disorders	0	(0.00)	0	(0.00)	
**Psycho-emotional**	4	(17.4)	7	(10.4)	0.462
Aggressiveness	0	(0.00)	0	(0.00)	
Fear/Anxiety	3	(13.0)	6	(8.96)	0.688
Impaired adaptation	1	(4.35)	1	(1.49)	0.448
**Sociocultural**	1	(4.35)	0	(0.00)	0.256
Language barriers	1	(4.35)	0	(0.00)	0.256
Social exclusion	0	(0.00)	0	(0.00)	
Belief conflict	0	(0.00)	0	(0.00)	
Illiteracy	0	(0.00)	0	(0.00)	
Lack of caregiver	0	(0.00)	0	(0.00)	
**Developmental**	6	(26.1)	22	(32.8)	0.732
Older age	6	(26.1)	22	(32.8)	0.732
**Comorbidities/Complications**	22	(95.7)	65	(97.0)	1.000
Major chronic disease	14	(60.9)	42	(62.7)	1.000
Haemodynamic instability	20	(87.0)	59	(88.1)	1.000
Risk of haemorrhage	0	(0.00)	0	(0.00)	
Communication disorders	0	(0.00)	0	(0.00)	
Urinary or faecal incontinence	1	(4.35)	1	(1.49)	0.448
Vascular fragility	0	(0.00)	1	(1.49)	1.000
Postural limitation	0	(0.00)	1	(1.49)	1.000
Involuntary movements	0	(0.00)	0	(0.00)	
Extreme weight	2	(8.70)	4	(5.97)	0.643
Dehydration	0	(0.00)	0	(0.00)	
Oedema	0	(0.00)	3	(4.48)	0.567
Uncontrolled pain	5	(21.7)	36	(53.7)	0.016
Transmissible infection	1	(4.35)	1	(1.49)	0.448
Immunosuppression	0	(0.00)	0	(0.00)	
Anatomical-functional disorders	0	(0.00)	2	(2.99)	1.000
Abbreviations: CCIFs, Care Complexity Individual Factors; EPLOS, Expected Length of Stay; SD, Standard Deviation.					

## Appendix F. Sensitivity Analysis Excluding Postoperative ERAS Adherence Items

	**ALL**	**Non-Adherent**	**Adherent**	**OR** **(95% CI)**	***p*-Value**
	*N* = 90	*N* = 24	*N* = 66		
**Postoperative Length of Stay (PLOS), median [Q1; Q3]**	5.00 [4.00; 8.00]	5.00 [4.00; 8.00]	5.00 [4.00; 7.00]	0.99 [0.93; 1.05]	0.484
**EPLOS compliance (%)**	23 (25.6%)	5 (20.8%)	18 (27.3%)	1.40 [0.47; 4.82]	0.729
**Complications, n (%)**	9 (10.0%)	4 (16.7%)	5 (7.58%)	0.41 [0.10; 1.89]	0.240
	ERAS adherence was recalculated excluding postoperative adherence items to minimise potential circularity between recovery-related variables and postoperative outcomes. Values are presented as median (IQR) or number (%), as appropriate. Odds ratios (OR) with 95% confidence intervals are unadjusted and derived from bivariate analyses; they should be interpreted cautiously. EPLOS: Expected postoperative length of stay.				
